# Inhibition of Influenza A Virus Replication by TRIM14 via Its Multifaceted Protein–Protein Interaction With NP

**DOI:** 10.3389/fmicb.2019.00344

**Published:** 2019-02-26

**Authors:** Xiangwei Wu, Jingfeng Wang, Shanshan Wang, Fei Wu, Zhigao Chen, Chunfeng Li, Genhong Cheng, F. Xiao-Feng Qin

**Affiliations:** ^1^Institute of Systems Medicine, Chinese Academy of Medical Sciences and Peking Union Medical College, Suzhou, China; ^2^Center for Systems Medicine, Institute of Basic Medical Sciences, Chinese Academy of Medical Sciences and Peking Union Medical College, Beijing, China; ^3^Department of Pathology, Institute of Precision Medicine, Jining Medical University, Jining, China

**Keywords:** TRIM14, influenza A virus, PRYSPRY domain, NP, protein–protein interaction

## Abstract

Influenza A virus (IAV) is a worldwide ongoing health threat causing diseases in both humans and animals. The interaction between IAV and host is a dynamic and evolving process that influences the pathogenicity and host specificity of the virus. TRIM14, a member of tripartite motif (TRIM) family, has been demonstrated to possess a strong capability of regulating type I interferon and NF-κB induction in host defense against viral infection. In this study, we found that TRIM14 could restrict the replication of IAV in a type I interferon and NF-κB independent manner. Mechanistically, different domains of TRIM14 could selectively interact with the viral nucleoprotein (NP), resulting in disparate influences on the RNP formation and viral replication. In particular, the PRYSPRY domain of TRIM14 exhibited a potent inhibitory activity on NP protein stability and IAV replication. On the contrary, the ΔS2 domain could rather antagonize the function of PRYSPRY domain and promote the IAV RNP formation by stabilizing NP. At the biochemical level, TRIM14-NP interaction could induce the K48-linked ubiquitination and proteasomal degradation of NP. Moreover, due to the rapid degradation of newly synthesized NP, TRIM14 could effectively block the translocation of NP from cytoplasm to nucleus thus further restrain the propagation of IAV in host cells. Taken together, our study has unraveled a previously unknown mechanism of TRIM14 mediated inhibition on RNP formation and influenza virus replication, and provides a new paradigm of complex and multifaceted host–pathogen interaction between ISG and viral protein.

## Introduction

Influenza A viruses, as major causative agents of respiratory infection, are responsible for at least 500,000 deaths worldwide each year (Medina and Garcia-Sastre, 2011; Petrova and Russell, 2017). The genome of IAV is composed of eight single stranded negative sense RNAs encoding 13 proteins. The viral ribonucleoprotein complex (vRNP) is composed of an RNA-dependent RNA polymerase complex (RdRp), a viral RNA and multiple NP. The polymerase complex consists of polymerase basic 2 (PB2), PB1 and polymerase acidic (PA). NP is a major structural protein that binds vRNA and cRNA to form the template of replication and transcription, and also interacts with the PB1 and PB2 to facilitate polymerization of RNP complex (Portela and Digard, 2002; Gabriel et al., 2008; Hu et al., 2017). The interaction of viral RNP components with cellular factors may determine the host specificity of IAV. However, only a few cellular factors which interact with the components of RNP have been illustrated so far (Mayer et al., 2007; Konig et al., 2010). For example, the importin-α2 recognizes nuclear localization signal peptides of NP thereby mediates the nuclear import of vRNP and NP (Wang et al., 1997; Cros et al., 2005). On the other hand binding of HSP90 to PB2 has been reported to promote the viral polymerase activity (Momose et al., 2002).

In response to influenza virus infection, the host immune system is often activated to prevent viral replication (Schneider et al., 2014). The viral RNA in infected cells is recognized by pattern recognition receptors (PRRs), resulting in the synthesis and secretion of type I interferons (IFNs). Type I IFNs subsequently induce the expression of a battery of downstream genes broadly known as IFN-stimulated genes (ISGs) which leads to an antiviral state effectively blocking viral replication (Katze et al., 2002; Pestka et al., 2004; Sadler and Williams, 2008; Shapira et al., 2009; Garcia-Sastre, 2011; Pothlichet et al., 2013; Schneider et al., 2014; Schoggins et al., 2014). Most members of TRIM family belong to ISGs and are involved in both innate and adaptive immunity against virus infection (Nisole et al., 2005; Ozato et al., 2008). It has been reported that TRIM5α can potently restrict retrovirus infection including HIV-1 (Towers, 2007). Similarly TRIM6 can inhibit hepatitis B virus (HBV) RNA transcription through interacting with HBV core promoter (Zhang et al., 2013). While TRIM19 has a broad activity in restraining the replication of various viruses including Ebola virus (EBOV), herpes simplex virus (HSV-1), lymphocytic choriomeningitis virus (LCMV) and IAV (Nisole et al., 2005). In addition, TRIM22 has been shown to play a role in restricting different DNA or RNA viruses, such as IAV, HBV, and encephalomyocarditis virus (EMCV) (Eldin et al., 2009; Gao et al., 2009; Di Pietro et al., 2013).

As a typical ISG protein, TRIM14 has been reported being able to activate type I IFN signaling through stabilizing cGAS, in which TRIM14 recruits USP14 to cleave K48-linked ubiquitination of cGAS. Besides, TRIM14 also involves in assembling WHIP-TRIM14-PPP6C complex to facilitate RIG-I-MAVS interaction as well as bridging NEMO to MAVS (Zhou et al., 2014; Chen et al., 2016; Tan et al., 2017). In our previous work, we showed that TRIM14 could directly inhibit hepatitis C virus (HCV) infection by promoting the degradation of the viral NS5A protein (Wang et al., 2016). However, its role in IAV infection has not been investigated. In this work, we have identified that different domains of TRIM14 could exert distinct effects on IAV RNP formation through multifaceted interaction with NP, thus revealing a direct anti-IAV mechanism of TRIM14.

## Results

### TRIM14 Inhibits IAV Replication Independent of Interferon and NF-κB Pathways

**Figure 1:**
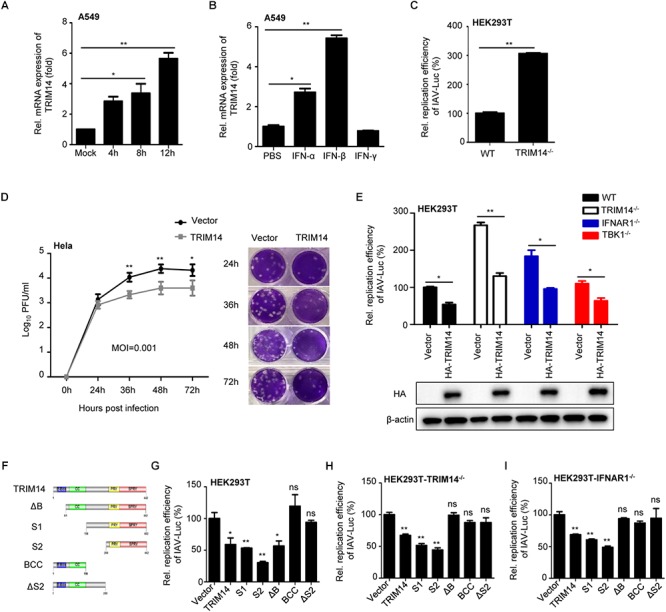
TRIM14 suppresses IAV replication independent of IFN-β and NF-κB pathway. **(A)** A549 cells were infected with WSN at an MOI of 5. Real-time PCR analysis of TRIM14 mRNA for the indicated time points. **(B)** Real-time PCR analysis of TRIM14 mRNA expression in A549 cells treated by 1000 IU IFN-α, IFN-β or IFN-γ. **(C)** HEK293T and TRIM14^-/-^ HEK293T cells were infected with IAV-Luc at an MOI of 0.01. The luciferase activity of supernatant was detected at 36 h post IAV-Luc infection. **(D)** HeLa cells were transfected with HA-TRIM14 or vector. Then cells were infected with WSN at an MOI of 0.001 at 24 h post transfection. Viral titers were measured by plaque assay for the indicated time points. The images of plaque assay were shown on the right. **(E)** Luciferase activity of IAV-Luc in HEK293T, TRIM14^-/-^ HEK293T, IFNAR1^-/-^ HEK293T and TBK1^-/-^ HEK293T cells transfected with TRIM14 expression plasmids. The immunoblot analysis of TRIM14 was shown below. **(F)** Schematic diagram of TRIM14 and TRIM14 mutants. **(G–I)** Luciferase activity of IAV-Luc in HEK293T **(G)**, TRIM14^-/-^ HEK293T **(H)** or IFNAR1^-/-^ HEK293T **(I)** cells transfected with expression vectors of TRIM14 or TRIM14 mutants. Data are presented as mean ± SEM, ^∗^*p* < 0.05; ^∗∗^*p* < 0.01; ns, not significant.

In order to identify the role of TRIM14 in controlling IAV infection, we first analyzed TRIM14 expression in human lung carcinoma cell line A549 infected with IAV or stimulated with type-I or II interferon. We found that IAV infection upregulated the expression of endogenous TRIM14 at the mRNA level (Figure 1A) comparable as IFN-β (Figure 1B). This confirmed that TRIM14, as an ISG, could be induced by IAV infection.

To investigate the functional significance of TRIM14 in restricting IAV replication, we generated TRIM14 knockout (KO) HEK293T cells by using CRISPR/Cas9 system. Depletion of TRIM14 protein expression in TRIM14^-/-^ cells was validated by Western blot analysis (Supplementary Figure S1A), and the relative mRNA expression of ISGs with poly I:C stimulation also confirmed the functional deficiency of TRIM14 in those KO cells (Supplementary Figure S1B). To directly measure the effect of TRIM14 KO on IAV replication, an engineered replication-competent IAV expressing luciferase reporter gene (IAV-Luc) was used (Pan et al., 2013). The wildtype HEK293T and TRIM14^-/-^ HEK293T cells were infected with IAV-Luc at an MOI of 0.01. The luciferase activity in the supernatant was measured at 36 h post infection. We observed that the IAV-Luc replication activity was enhanced ∼3-fold in TRIM14^-/-^ HEK293T cells compared with wildtype HEK293T cells (Figure 1C). The result thus indicated that TRIM14 deficiency could promote the replication of IAV. To further confirm the function of TRIM14 on inhibiting natural IAV replication, we transfected plasmid expressing TRIM14 or an empty vector as a control in HeLa cells. Then cells were infected with WSN at an MOI of 0.001 and 0.01 (Figure 1D and Supplementary Figure S1C, respectively). Viral titers were determined for the indicated time points by plaque assay. The results showed that overexpression of TRIM14 could potently inhibit the replication of WSN.

It has been reported that TRIM14 promotes RIG-I-MAVS-mediated type I IFN signaling stimulated by RNA virus infection. We therefore examined whether the inhibition of IAV replication by TRIM14 was due to activation of type I IFN signaling. As TBK1 is the common downstream molecule of RIG-I-MAVS and cGAS-STING pathway, we constructed TBK1 knockout cells to suppress the secretion of IFN induced by TRIM14. The genomic DNA sequence analysis of TBK1 knockout cells was shown in Supplementary Figure S1D and functional validation was shown in Supplementary Figure S1F. Furthermore, we also generated IFNAR1 knockout cells to block type I IFN response. The sequence alignment analysis was shown in Supplementary Figure S1E and functional validation of IFNAR1^-/-^ cells was shown in Supplementary Figure S1F. Next we detected the IAV-Luc replication in IFNAR1^-/-^ HEK293T and TBK1^-/-^ HEK293T cells after TRIM14 overexpression. We found that TRIM14 still inhibited IAV replication in the condition of IFNAR1 deficiency or TBK1 deficiency (Figure 1E). The results strongly suggest that there is an IFN-independent mechanism involving in TRIM14 inhibiting IAV replication. Meanwhile, we constructed different TRIM14 mutants (ΔB, S1, S2, BCC and ΔS2) (Figure 1F) to identify which domain is responsible for restricting IAV replication. We detected the activity of TRIM14 and TRIM14 mutants on activating IFN-β-luc and NF-κB-luc reporters. These results confirmed that TRIM14 mutants had much lower activity on enhancing activation of IFN-β (Supplementary Figure S1G), NF-κB and ISRE (Wang et al., 2016) than full-length TRIM14 protein. Next, plasmids expressing HA-tagged TRIM14 or TRIM14 mutants were transfected in HEK293T, TRIM14^-/-^ HEK293T or IFNR1^-/-^ HEK293T cells. Cells were infected with IAV-Luc at 24 h post transfection. We observed that the relative replication activities of IAV-Luc were impaired when transfected with plasmids containing PRYSPRY domain which has little capability to induce IFN-β and NF-κB (Figure 1G–I). These suggest that PRYSPRY domain indeed could work independent of the wild type TRIM14 protein in restricting IAV replication. All of above data demonstrate that the mechanism of TRIM14 inhibiting IAV replication is not through NF-κB or IFN-β pathway.

### TRIM14 Restricts Viral RNP Formation

**Figure 2:**
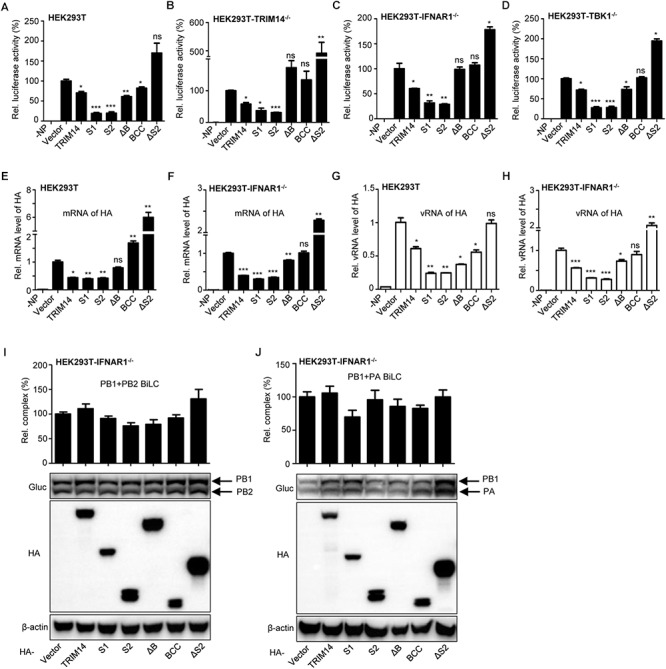
TRIM14 impairs the formation of viral RNP complex. **(A)** IAV RNP formation was determined in HEK293T cells transfected with plasmids for PB1, PB2, PA, NP and vRNA-luciferase reporter plasmid as well as plasmids expressing TRIM14 or TRIM14 mutants. Negative control was transfected with plasmids of the RNP reconstitution reporter system except for NP. Luciferase activity was detected at 24 h post transfection. **(B–D)** The luciferase activity of RNP formation was performed in TRIM14^-/-^ HEK293T **(B)**, IFNAR1^-/-^ HEK293T **(C)** or TBK1^-/-^ HEK293T **(D)** cells. **(E–H)** HEK293T or IFNAR1^-/-^ HEK293T cells were co-transfected with RNP reconstitution plasmids (PB1, PB2, PA, NP and pPOLI-HA) and TRIM14 or TRIM14 mutants. Total RNA was extracted at 24 h after transfection for quantitative PCR of HA mRNA **(E,F)** or HA vRNA **(G,H)**. **(I,J)** BiLC assay of the interaction between PB1 and PB2 **(I)**, or PB1 and PA **(J)**. PB1 was fused to GlucN, PB2 and PA were fused to GlucC. Plasmids expressing PB1-GlucN and PB2-GlucC **(I)**, or GlucN-PB1 and PA-GlucC **(J)** were co-transfected with TRIM14 or TRIM14 mutants in IFNAR1^-/-^ HEK293T cells. The relative luminescence units (RLU) were detected by a microplate reader at 36 h post transfection. The expression of proteins was detected by immunoblot analysis. Data are presented as mean ± SEM, ^∗^*p* < 0.05; ^∗∗^*p* < 0.01; ^∗∗∗^*p* < 0.001; ns, not significant.

The synthesis efficiency of viral RNA, which is essential for virus propagation and pathogenicity, is dependent on the formation of viral RNP complex (Klumpp et al., 1997; Te Velthuis et al., 2016). So we utilized a reporter-based viral RNP reconstitution mini-genome system to determine whether the inhibition on IAV replication by TRIM14 results from restricting viral RNP formation (the schematic diagram of IAV RNP reconstitution system was shown in Supplementary Figure S2A.) HEK293T cells were co-transfected with plasmids expressing TRIM14 or TRIM14 mutants and a viral RNP reconstitution reporter system. The viral RNP reconstitution reporter system contained plasmids expressing PB1, PB2, PA, NP and a reporter vector expressing minus-sense luciferase RNA flanked by UTR from NP segment of WSN. We found that the relative quantity of functional RNP complex was obviously reduced when transfected with plasmids containing PRYSPRY domain (Figure 2A). The S1, S2 mutants demonstrated the most profound defect with relative luciferase activities were reduced to under 50%. Similar results were observed when experiments were repeated in the TRIM14^-/-^ HEK293T, IFNR1^-/-^ HEK293T and TBK1^-/-^ HEK293T cells (Figure 2B–D). These results indicate that generally decreased activity resulted from TRIM14 does not depend on stimulating IFN-β and NF-κB. To directly assess the consequences of defective RNP complex, we measured the products of vRNA replication (vRNA) and transcription (mRNA) using quantitative real-time PCR. Decreased levels of mRNA and vRNA were detected in the presence of PRYSPRY domain, respectively (Figure 2E–H), which were consistent with the levels of luciferase activity detected in the mini-genome assay. These indicate that TRIM14 PRYSPRY domain could impair the formation of IAV RNP complex and viral replication and transcription.

Then we investigated the effect of TRIM14 on the assembly of IAV polymerase by a BiLC-based reporter system as reported before (Li et al., 2017). We found no statistically inhibitory effect on PB1 and PB2 interaction as well as PB1 and PA interaction when TRIM14 or TRIM14 mutants were overexpressed in IFNR1^-/-^ HEK293T cells (Figure 2I,J). These results imply that TRIM14 has no effect on the RdRp complex formation and polymerase assembly.

### TRIM14 Interacts With NP

**Figure 3:**
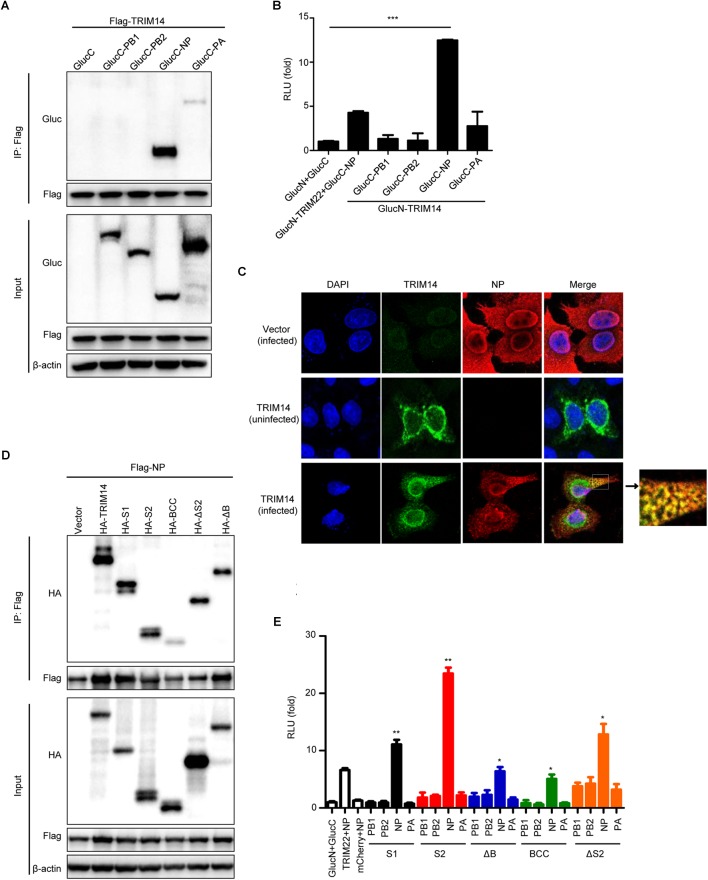
TRIM14 interacts with NP. **(A)** CoIP and immunoblotting of extracts of HEK293T cells co-transfected with Flag-TRIM14 and GlucC-X (X = PB1, PB2, NP, PA). Cells were collected at 36 h after transfection for immunoprecipitation and immunoblot analysis. **(B)** Interactions between TRIM14 and IAV RNP components screened by BiLC assay. Plasmids expressing TRIM14-GlucN, GlucN-TRIM14, X-GlucC and GlucC-X were co-transfected in HEK293T cells. The RLU were detected by a microplate reader at 36 h post transfection. The interaction between TRIM22 and NP was determined as positive control. **(C)** The co-localization of TRIM14 and NP. HeLa cells were transfected with vector (top) or HA-TRIM14 (bottom) then infected with WSN (MOI of 1) at 24 h after transfection. Cells were fixed for confocal microscopy at 12 h post infection. As a control, cells were transfected with HA-TRIM14 without WSN infection then fixed for confocal microscopy (middle). The red fluorescence showed the staining of NP and green fluorescence showed the staining of TRIM14. **(D)** CoIP and immunoblotting of extracts of HEK293T cells co-transfected with Flag-NP and HA-TRIM14 or TRIM14 mutants. **(E)** Interactions between TRIM14 mutants and IAV RNP components screened by BiLC assay. Plasmids expressing TRIM14 mutants-GlucN, GlucN-TRIM14 mutants, X-GlucC and GlucC-X were co-transfected in HEK293T cells. The RLU were detected by a microplate reader at 36 h post transfection. Data in **(B,E)** are presented as mean ± SEM, ^∗^*p* < 0.05; ^∗∗^*p* < 0.01; ^∗∗∗^*p* < 0.001.

To identify the underlying mechanism involved in the inhibition of IAV replication by TRIM14, we investigated the interaction between TRIM14 and RNP components through Co-IP assay. Flag-tagged NP and HA-tagged TRIM14 were co-expressed in HEK293T cells. The result showed that TRIM14 obviously interacted with NP and showed weak interaction with PA (Figure 3A). The interaction between TRIM14 and NP was further confirmed by BiLC assay (Figure 3B). In addition, HeLa cells were transfected with HA-TRIM14 or vector then infected with WSN at 24 h post transfection. Cells were fixed for immunofluorescence at 12 h after infection. The co-localization of TRIM14 and NP in the cytoplasm was evidently revealed by confocal microscopy (Figure 3C, bottom) compared with the control without IAV infection (Figure 3C, middle). As a further support, we also detected the interaction of TRIM14 mutants and NP by Co-IP assay. CoIP assay showed that TRIM14 and TRIM14 mutants interacted with NP while BCC only had weak interaction with NP (Figure 3D). In a components study, we examined the interaction of TRIM14 mutants and RNP components by BiLC assay. Results showed that all of TRIM14 mutants presented remarkable interaction with NP rather than other proteins (PB1, PB2, PA) (Figure 3E). These findings suggest that TRIM14 could interact with NP through multiple distinct domains.

### TRIM14 PRYSPRY Domain Enhances the Degradation of NP

**Figure 4:**
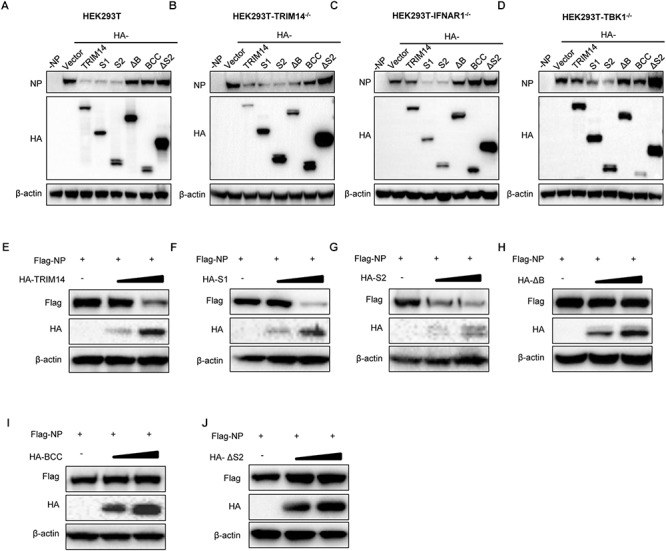
TRIM14 PRYSPRY domain promotes the degradation of NP. **(A–D)** Immunoblot analysis of extracts of HEK293T **(A)**, TRIM14^-/-^ HEK293T **(B)**, IFNAR1^-/-^ HEK293T **(C)** or TBK1^-/-^ HEK293T **(D)** cells co-transfected with RNP reconstitution system and TRIM14 or TRIM14 mutants. The relative quantifications of NP were shown in Supplementary Figures S3A–D. **(E–J)** Immunoblot analysis of extracts of HEK293T cells co-transfected with Flag-NP (200 ng) and increasing amounts (0 ng, 200 ng, 400 ng) of HA-TRIM14 **(E)**, HA-S1 **(F)**, HA-S2 **(G)**, HA-ΔB **(H)**, HA-BCC **(I)**, and HA-ΔS2 **(J)**. The relative quantifications of NP were shown in Supplementary Figures S3E–J.

To further explore how TRIM14 regulates NP through their multifaceted interaction, TRIM14 or TRIM14 mutants were transfected together with the RNP reconstitution system in HEK293T, TRIM14^-/-^ HEK293T, IFNR1^-/-^ HEK293T and TBK1^-/-^ HEK293T cells. Immunoblot analysis showed that the protein of NP was destroyed in the presence of PRYSPRY domain (Figure 4A–D). We also detected the expression of NP after infected with IAV-Luc. As expected, the expression of NP was diminished post TRIM14 overexpression (Supplementary Figure S3K). To confirm the decreasing protein expression of NP mediated by PRYSPRY domain, HEK293T cells were co-transfected with TRIM14 or TRIM14 mutants and fixed amount of NP expressing plasmids. The results confirmed that S1, S2 mutants led to a significant loss of NP while BCC and ΔS2 mutants failed to promote NP degradation (Figure 4E–J). We conclude that TRIM14 PRYSPRY domain mediates the degradation of NP.

### TRIM14 ΔS2 Suppresses S2-NP Interaction and Stabilizes the Expression of NP

**Figure 5:**
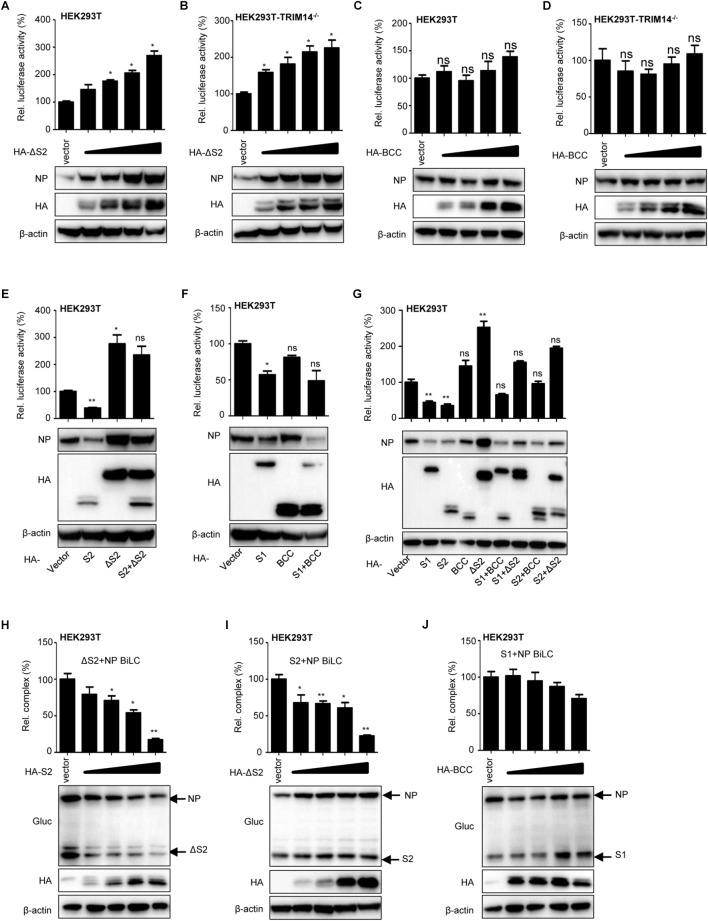
TRIM14 ΔS2 mutant induces accumulation of NP. **(A,B)** IAV RNP formation and immunoblot analysis of extracts of HEK293T **(A)** or TRIM14^-/-^ HEK293T **(B)** cells co-transfected with RNP reconstitution reporter system and increasing amounts (vector, 25 ng, 50 ng, 100 ng, 200 ng) of HA-ΔS2. **(C,D)** IAV RNP formation and immunoblot analysis of extracts of HEK293T **(C)** or TRIM14^-/-^ HEK293T **(D)** cells co-transfected with RNP reconstitution reporter system and increasing amounts (vector, 25 ng, 50 ng, 100 ng, 200 ng) of HA-BCC. **(E)** IAV RNP formation and immunoblot analysis of HEK293T cells co-transfected with RNP reconstitution reporter system and expression plasmids of S2, ΔS2, S2+ΔS2. **(F)** IAV RNP formation and immunoblot analysis of HEK293T cells co-transfected with RNP reconstitution reporter system and expression plasmids of S1, BCC, S1+BCC. **(G)** IAV RNP formation and immunoblot analysis of HEK293T cells co-transfected with RNP reconstitution reporter system and expression plasmids of S1, S2, BCC, ΔS2, S1+BCC, S1+ΔS2, S2+BCC, S2+ΔS2. **(H)** BiLC assay of the interaction between ΔS2 and NP. Plasmids expressing ΔS2-GlucN, GlucN-ΔS2, NP-GlucC, GlucC-NP were co-transfected with increasing amounts (vector, 100 ng, 200 ng, 300 ng, 400 ng) of HA-S2 in HEK293T cells. The luciferase activity was detected at 36 h post transfection. The expression of proteins was detected by immunoblot analysis. **(I)** Plasmids expressing S2-GlucN, GlucN-S2, NP-GlucC, GlucC-NP were co-transfected with increasing amounts (vector, 100 ng, 200 ng, 300 ng, 400 ng) of HA-ΔS2 in HEK293T cells. The luciferase activity was detected at 36 h post transfection. The expression of proteins was detected by immunoblot analysis. **(J)** Plasmids expressing S1-GlucN, GlucN-S1, NP-GlucC, GlucC-NP were co-transfected with increasing amounts (vector, 100 ng, 200 ng, 300 ng, 400 ng) of HA-BCC in HEK293T cells. The luciferase activity was detected at 36 h post transfection. The expression of proteins was detected by immunoblot analysis. Data are presented as mean ± SEM. ^∗^*P* < 0.05, ^∗∗^*P* < 0.01, ns, not significant.

More intriguingly, we observed that the viral RNP formation and intracellular protein level of NP were increased with overexpression of ΔS2 mutant in the RNP reconstitution system. We speculated that the other domain of TRIM14 could regulate IAV replication except for PRYSPRY domain. Thus, fixed amount of RNP expressing plasmids with increasing amounts of ΔS2 expressing plasmid were co-transfected in HEK293T cells for immunoblot analysis and luciferase activity of RNP formation. Remarkably, increasing amounts of ΔS2 resulted in the increase accumulation of NP expression and RNP formation (Figure 5A,B). We also overexpressed BCC as control and found that BCC had no significant effect on the expression of NP (Figure 5C,D). These results indicate that other domain on ΔS2 mutant could stabilize NP expression.

Then we examined the expression of NP and formation of RNP after transfecting S2, ΔS2, S2+ΔS2 or S1, BCC, S1+BCC in HEK293T and TRIM14^-/-^ HEK293T cells (Figure 5E and Supplementary Figure S3L). When S2+ΔS2 was overexpressed, the protein of NP and formation of RNP complex were higher than overexpression of S2 but lower than overexpression of ΔS2. But the same phenomenon was not observed when overexpression of S1+BCC (Figure 5F and Supplementary Figure S3M). The effects of S1+ΔS2 on the expression of NP and formation of RNP complex were further examined as well. The data showed that ΔS2 could still enhance the level of intracellular NP with co-expression of S1. But only BCC had no potency to control the degradation of NP by PRYSPRY domain (Figure 5G). These suggest that TRIM14 ΔS2 mutant restricts the degradation of NP resulted from PRYSPRY domain.

Furthermore, BiLC assay was also used to investigate the influence of S2 on ΔS2-NP interaction or ΔS2 on S2-NP interaction. We observed that the interaction between ΔS2 and NP was impaired with overexpression of S2 (Figure 5H). The inhibitory effect of S2 on ΔS2-NP interaction was probably due to the degradation of NP resulted from overexpression of S2. Likewise, the interaction between S2 and NP was inhibited by ΔS2 (Figure 5I). However, we didn’t find the inhibitory effect of BCC on S1-NP interaction (Figure 5J). In addition, we also detected the interaction between S2 and ΔS2 mutants (Supplementary Figure S3N). Therefore, we supposed that ΔS2 could restrict the interaction of S2 and NP through interplaying with NP or S2 (Supplementary Figure S2C).

Collectively, these results demonstrate that TRIM14 ΔS2 mutant enhances the level of intracellular NP, which might offset the inhibitory effect of PRYSPRY domain.

### TRIM14 Promotes the Proteasome Mediated Degradation of NP and Restrain the Translocation of NP to Nucleus

**Figure 6:**
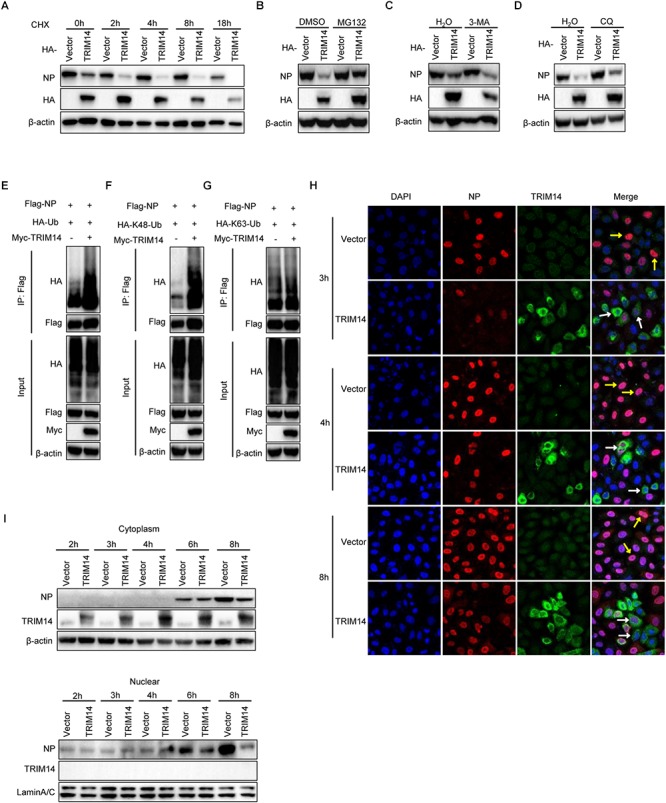
TRIM14 induces the destabilization of NP and inhibits the translocation of NP to nucleus. **(A)** HEK293T cells were co-transfected with RNP reconstitution system and TRIM14. Cells were treated by 100 μM CHX at 24 h after transfection and collected at the time points shown for immunoblot analysis. **(B–D)** Immunoblot analysis of extracts of HEK293T cells co-transfected with RNP reconstitution system and TRIM14. At 24 h post transfection, cells were treated with 8 μM MG132 **(B)**, 5 mg/ml 3-MA **(C)** or 20 μM CQ **(D)** for 8 h, 8 h, 20 h. **(E)** Immunoblot analysis of extracts of HEK293T cells co-transfected with plasmids of Flag-NP, HA-Ubiquitin, Myc-vector or Myc-TRIM14 followed by immunoprecipitation with anti-Flag beads. **(F,G)** Immunoblot analysis of extracts of HEK293T cells co-transfected with plasmids of Flag-NP, Myc-TRIM14, HA-K48-Ubiquitin **(F)** or HA-K63-Ubiquitin **(G)** followed by immunoprecipitation with anti-Flag beads. **(H)** HeLa cells were transfected with HA-TRIM14 or vector then infected with WSN at an MOI of 1 at 24 h after transfection. Cells were fixed for confocal microscopy at 3 h, 4 h, and 8 h post infection. The red fluorescence showed the staining of NP and green fluorescence showed the staining of TRIM14. **(I)** HeLa cells were transfected with HA-TRIM14 or vector then infected with WSN (MOI of 1) at 24 h after transfection. Cells were harvested for the indicated time points after infection for immunoblot analysis.

To delineate biochemical mechanisms for NP destabilization by TRIM14, we tested the degradation of NP by TRIM14 in the presence of translation inhibitor cycloheximide (CHX). HEK293T cells were co-transfected with plasmids of TRIM14 and RNP reconstitution system. Then cells were treated with 100μM CHX and harvested for immunoblot analysis. The results showed that TRIM14 still strongly degraded NP with the treatment of CHX (Figure 6A). This implied that TRIM14 could promote the destabilization of NP. To further determine the major pathway involved in the degradation of NP, we examined the degradation of NP with treatment of proteasome inhibitor MG132 or autophagic-sequestration inhibitor 3-methyladenine (3-MA) or lysosomal-acidification inhibitor chloroquine (CQ). The results showed that the degradation of NP was canceled in the presence of MG132 but not 3-MA or CQ (Figure 6B–D). These indicate that ubiquitin-proteasome system is required for degradation of NP by TRIM14. To address this, we detected the ubiquitination of NP in HEK293T cells co-transfected with Flag-NP, HA-Ubiquitin and Myc-TRIM14 or vector. Immunoprecipitation and immunoblot analysis showed that TRIM14 induced the ubiquitination of NP (Figure 6E). Furthermore, we observed that TRIM14 enhanced the K48-linked ubiquitination of NP rather than K63-linked ubiquitination (Figure 6F,G), which was consistent with early data (Figure 6B), as K48-linked ubiquitination normally facilitated proteasomal degradation of protein. Collectively, these findings suggest that TRIM14 mediates the degradation of NP through ubiquitin- proteasome pathway.

To investigate how TRIM14 influence IAV replication through promoting degradation of NP in the context of virus infection, HA-TRIM14 or vector were transfected in HeLa cells. Cells were infected with WSN (MOI = 1) at 24 h post transfection. Then cells were fixed in different time points for immunofluorescence and also collected for immunoblot analysis to measure the protein of NP in the nucleus and cytoplasm fractions. The immunofluorescence assay showed that almost all cells with TRIM14 overexpression only had weak or no NP signal in the nucleus (indicated by white arrowheads). In contrast, the control cells transfected with the empty vector had strong NP staining in the nucleus (indicated by yellow arrowheads) (Figure 6H). Consistent with the co-focal microscopy result, our immunoblot analyses showed that NP was diminished in the cytoplasm and also markedly decreased in the nucleus of the cells transfected HA-TRIM14 expression vector compared to the empty vector controls (Figure 6I). Therefore, we inferred that TRIM14 could interact with the newly synthesized NP in the cytoplasm and degrade NP in a short time. This process thus might impart a strong effect on restricting the translocation of NP to nucleus leading to the reduced expression of NP in the nucleus.

In conclusion, our data indicate that TRIM14 could mediate the proteasome dependent degradation of NP, and rapid degradation of NP in cytoplasm result in an effective blockade for NP translocation to the nucleus in IAV infected host cells.

## Discussion

Extensive molecular analysis of host–pathogen interaction has brought great advances in our understanding of innate immune responses to influenza virus infection in recent years (Akira et al., 2006; Pichlmair et al., 2006; Seo et al., 2010; Barbalat et al., 2011; Iwasaki and Pillai, 2014; Tripathi et al., 2015). Several ISGs limit IAV infection by directly targeting pathways or functions required during virus life cycle. And every stage of the virus life cycle is a potential target for ISG intervention (Watanabe et al., 2010; Desai et al., 2014; Iwasaki and Pillai, 2014). For example, viperin restricts influenza virus release by blocking the formation of the lipid raft and inhibits viral budding (Wang et al., 2007). CH25H has a broad antiviral effect for enveloped viruses including influenza virus by interfering virus-host membrane fusion (Blanc et al., 2013; Liu et al., 2013). TRIM22 targets IAV NP for proteasomal degradation (Di Pietro et al., 2013). However, some other ISGs and their anti-viral mechanism remain to be investigated in detail. Here we demonstrated the biological significance and molecular mechanism of TRIM14 restricting IAV replication.

Recent studies reported that TRIM14 could inhibit DNA viruses by stabilizing cGAS to upregulate type I IFN signaling and restrict RNA viruses through regulating RIG-I-MAVS-mediated interferon pathway (Zhou et al., 2014; Chen et al., 2016; Tan et al., 2017). However, in our study, TRIM14 exhibited equal potency of inhibiting IAV replication in wild type HEK293T cells and IFNAR1 knockout as well as TBK1 knockout cells (Figure 1E). In particular, the replication of IAV was mostly restricted by PRYSPRY domain, while this domain had low potency to induce IFN-β and NF-κB. Thus we believed that it must exist other mechanism involving in TRIM14 inhibiting IAV replication apart from the induction of IFN-β and NF-κB pathways. Moreover, our previous study discovered that TRIM14 inhibits HCV by degrading NS5A protein (Wang et al., 2016). Based on these observations, we hypothesized that restriction of IAV by TRIM14 appeared mechanistically similar to the anti-HCV effects. Inspiringly, we observed a remarkable interaction between TRIM14 and NP (Figure 3).

Viral RNP consisting of the polymerase and NP coated vRNA is indispensable to viral replication and transcription (Herz et al., 1981; Klumpp et al., 1997; Coloma et al., 2009; Fodor, 2013). NP is an RNA binding protein that represents an important structural component of RNP and is essential for both replication and transcription of full-length RNA genome segments (Klumpp et al., 1997; Moeller et al., 2012; Fodor, 2013; Te Velthuis and Fodor, 2016). In this research, we performed the effect of TRIM14 on RNP formation with an RNP reconstitution reporter system. We found that TRIM14 definitely impaired the formation of functional RNP complex, especially PRYSPRY domain maintained a profound restriction on viral RNA synthesis and RNP formation among HEK293T, TRIM14^-/-^ HEK293T, IFNAR^-/-^ HEK293T and TBK1^-/-^ HEK293T cells (Figure 2). Further molecular mechanism studies found obvious interaction between TRIM14 and NP, which confirmed that TRIM14 could suppress IAV replication independent of interferon pathway (Figure 3).

Evolutionary signatures of positive selection on the PRYSPRY domain indicate that it is under strong selective pressure. This evolutionary perspective of innate cellular immunity has been proven very essential in characterizing and identifying ISGs that directly interact with pathogens (Rhodes et al., 2005; Sawyer et al., 2007; Duggal and Emerman, 2012). The PRYSPRY domain contains a putative interaction site which can bind to various target proteins to exert distinct functions. In previously reported studies, TRIM14 could recruit USP14 and interact with cGAS through PRYSPRY domain. In our present work, we also constructed S1 and S2 mutants containing PRYSPRY domain and detected significant inhibition on IAV replication by this domain and observed its interaction with NP (Figure 3D,E). Having shown the interplay of NP and TRIM14 mutants, we next detected NP destabilization induced by TRIM14 (Figure 4). Further experiments demonstrated that TRIM14 enhanced the K48-ubiquitination and proteasomal degradation of NP (Figure 6). As TRIM14 is not an E3 ubiquitin ligase without RING domain, we speculated that the manner by which TRIM14 chose the degradation pattern on NP was due to some indirect effect on NP such as recruiting an E3 ubiquitin ligase.

During viral replication, the mRNA of NP in the nucleus is exported to cytoplasm for translation. Then the protein of NP is translocated back to nucleus to form progeny RNP complex. The progeny RNPs are subsequently exported to the cytoplasm for virus packaging and budding (Medina and Garcia-Sastre, 2011). In this study, we found that the degradation of NP by TRIM14 led to effective inhibition of translocation of newly synthesized NP in the cytoplasm to the nucleus (Figure 6H,I). Besides, we observed the co-localization of TRIM14 and NP when progeny RNPs were exported to the cytoplasm (Figure 3C). Therefore, we supposed that TRIM14 interacted with the free NP newly synthesized and the oligomeric NP on RNP complex in the cytoplasm then rapidly degraded NP. The degradation of free NP contributed to the restriction of translocation of NP to nucleus, which resulted in very low expression of NP in the nucleus after IAV infection.

In addition, we noticed an increased formation of RNP complex and higher levels of NP protein when ΔS2 mutant was overexpressed (Figure 2, 4, 5). Further experiments demonstrated that overexpression of ΔS2 led to increased RNP formation and accumulation of NP. The protein expression of NP was also higher with S2+ΔS2 overexpression than S2 overexpression (Figure 5). Moreover, we observed the interaction between ΔS2 and S2 (Supplementary Figure S3N). Consequently, we considered that ΔS2 and S2 could competitively interplay with NP and exert opposite role on NP expression and IAV replication. Meanwhile, ΔS2 might interact with S2 and further suppress the decrease of NP resulted from PRYSPRY domain. But BCC had no similar effects. It’s well established that virus evolution could constantly generate new mechanism to evade host immunity. For instance, IAV NS1 could interact with the coil-coil domain of TRIM25 to inhibit TRIM25-mediated ubiquitination and activation of RIG-I and further facilitate the replication of IAV (Gack et al., 2009). Since TRIM14 is a multifunctional protein, we could not exclude the possibility that TRIM14 other domain interacts with IAV proteins to promote viral infection and negatively regulate the anti-IAV effect of TRIM14. Alternative interpretation is that the virus might have evolved a mechanism to evade PRYSPRY mediated host restriction through interaction with other adjacent domains of TRIM14 protein. This hypothesis would explain the observations that full-length TRIM14 presented less inhibitory activity on virus replication and NP protein expression than PRYSPRY domain. This remains to be further explored.

In summary, we have uncovered a novel mechanism of TRIM14 in restricting IAV replication with providing evidence that IAV NP interacts with multiple domains of TRIM14. Our study thus offers new insights into the biochemical role of TRIM14, emphasizing the need for better understanding the complex and multifaceted interaction between virus and host.

## Materials and Methods

### Cells and Virus

HEK293T, A549, HeLa, and MDCK cells were cultured in DMEM (Gibco) supplemented with 10% FBS (Gibco) and 2 mM L-glutamine (Gibco). All cells were maintained at 37°C with 5% CO_2_. IAV-Luc (H1N1/PR8) virus with luciferase reporter on NA gene was kindly provided by Dr. Chen Ling (Guangzhou Biomedicine and Health Institute, Chinese Academy of Sciences) and propagated in chicken eggs. Influenza A/WSN/33 (WSN) virus was amplified in MDCK cells in Opti-MEM with 1 μg/ml tosylsulfonyl phenylalanyl chloromethyl ketone (TPCK)-trypsin.

### Plasmids and Antibodies

Plasmids expressing TRIM14 and TRIM14 mutants have been described previously (Wang et al., 2016). The plasmids pcDNA-PB1, pcDNA-PB2, pcDNA-PA, and pcDNA-NP expressing influenza A/WSN/33 virus proteins as well as the vRNA-expressing plasmids pPOLI-NP-RT-Luc and pPOLI-HA were gifts from Dr. Tao Deng (Institute of Pathogen Biology, Chinese Academy of Medical Sciences). The full-length NP cDNA was cloned into the pCMV-Flag expression vector (Beyotime, China) to obtain Flag-NP fusion protein.

Rabbit anti-TRIM14 antibody was purchased from Aviva Systems Biology. Horseradish Peroxidase conjugated anti-Flag (M2) antibody was purchased from Sigma. Horseradish Peroxidase conjugated anti-HA antibody was from Roche. Mouse anti-NP antibody was purchased from Millipore. Rabbit anti-Gluc antibody was purchased from NEB. Mouse anti-c-myc and mouse anti-beta actin antibody were from Cell Signaling Technology. Rabbit anti-lamin A/C was from Proteintech. FITC conjugated anti-rabbit IgG antibody and Alexa Fluor^®^ 594 conjugated anti-mouse IgG antibody were purchased from ZSGB-BIO. DMSO, MG-132, 3-MA (3-methyladenine), CQ (chloroquine), CHX (cycloheximide) were purchased from Sigma.

### Knockout TRIM14/IFNAR1/TBK1 by CRISPR/Cas9 System

HEK293T cells cultured in 24-well plates were transfected with 500 ng plasmids expressing sgRNA and CRISPR/Cas9 by lipofectamine 3000 (Invitrogen). Cells were selected by 5 μg/ml puromycin at 36 h after transfection. Two days later, cells were seeded in 96 well plates for amplification. Then cells were harvested for verification by DNA sequencing or Western blot. The sgRNA of target genes are shown in Supplementary Materials and Methods.

### Quantitative Real-Time PCR

Total RNA was extracted using NucleoSpin RNA Plus kit (MACHEREY-NAGEL) and reverse-transcribed using the PrimeScript 1st-Strand cDNA Synthesis Kit (TaKaRa). Real-time PCR was performed using SYBR Green Master Mix (Roche). The relative mRNA expression was normalized to GAPDH. The fold modulation in gene expression was analyzed by 2^-ΔΔCt^ method. The primers of target genes are shown in Supplementary Materials and Methods.

### RNP Reconstitution Reporter System Assay

HEK293T cells cultured in 24-well plates were transfected with plasmids encoding PB2, PB1, PA and NP of WSN (50 ng each) along with 50 ng vRNA-luciferase reporter plasmid, and a *Renilla* luciferase plasmid (5 ng) as an internal control. Cells were harvested and lysed at 24 h after transfection. Reporter activity was analyzed with the Dual-Luciferase Reporter Assay system (Promega).

### IAV-Luc Infection

HEK293T cells were infected with IAV-Luc at an MOI of 0.01 with 0.1% TPCK-trypsin (1 μg/ml) in DMEM. Thirty-six hours post-infection, the viral titers were measured by detecting the luciferase activity of supernatant by a microplate reader. The experiment of IAV-Luc infection was under the same condition and safety level with WSN.

### Plaque Assay

MDCK cells were seeded in a 12-well plate for 14–16 h. Cells were washed with PBS and infected with WSN for 1 h at 37°C. Viral inoculations were aspirated and replaced with DMEM containing 1% low melting agarose and 1% BSA. Viral plaques were developed at 72 h.

### BiLC Reporter Assay

In the BiLC assay, the Gaussia luciferase protein was divided into two parts GlucN (17–109) and GlucC (110–185), which mediated protein–protein interactions. TRIM14 and TRIM14 mutants were fused with GlucN, while RNP proteins (PB1, PB2, PA and NP) were connected to GlucC to produce GlucC-X/ X-GlucC (X = PB1, PB2, PA or NP) fusion proteins (Supplementary Figure S2B).

The GlucN-TRIM14 or TRIM14 mutants, TRIM14 or TRIM14 mutants-GlucN, GlucC-X (X = PB1, PB2, PA or NP) and X-GlucC plasmids (50 ng each) were co-transfected in HEK293T cells seeded in a 96-well plate by PEI (Sigma). Cells were harvested and lysed at 36 h after transfection. Reporter activity was analyzed with the Renilla Luciferase Assay system (Promega).

### Immunofluorescence

HeLa cells were transfected with HA-TRIM14 or a vector then cells were infected with WSN at an MOI of 1. After infection, cells were fixed with 4% paraformaldehyde for 10 min at 4°C and permeated with 0.5% Trion X-100 for 15 min at room temperature. Cells were stained by mouse anti-NP and rabbit anti-TRIM14 antibodies followed by secondary antibodies of FITC conjugated anti-rabbit IgG antibody and Alexa Flour^®^ 594 conjugated anti-mouse IgG antibody. Nuclear was stained with DAPI for 5 min. Images were acquired with 63 × oil immersion objective of Leica TCSSP8 confocal microscope.

### Immunoprecipitation and Immunoblotting

HEK293T cells were cotransfected with Flag-NP and HA-TRIM14 or TRIM14 mutants. Cells were harvested at 36 h after transfection and were lysed with RIPA buffer, followed incubation overnight at 4°C with the anti-Flag beads (Sigma). Beads were washed five times with RIPA buffer and boiled with 2 × SDS-loading buffer. Immunoprecipitates were resolved by SDS-PAGE gel electrophoresis then transferred to PVDF membranes, further incubated with appropriate antibodies.

### Statistical Analysis

The results were presented as the mean ± SEM from at least three independent experiments with GraphPad Prism 5 software. A Student *t*-test was used to determine statistical significance. *P*-value < 0.05 was considered to be significant.

## Data Availability

All datasets generated for this study are included in the manuscript and the Supplementary Files.

## Author Contributions

XW performed most of the experiments, analyzed the results, and wrote the manuscript. JW, SW, FW, CL, and GC provided essential reagents and methods. ZC performed the supporting experiments. FQ conceived and supervised the study and wrote the manuscript.

## Conflict of Interest Statement

The authors declare that the research was conducted in the absence of any commercial or financial relationships that could be construed as a potential conflict of interest.

## Abbreviations

BiLC: bimolecular luminescence complementation
cGAS: cyclic GMP-AMP synthase
CoIP: co-immunoprecipitation
IAV: influenza A virus
IFNAR1: interferon alpha and beta receptor subunit 1
ISG: interferon stimulated gene
MOI: multiplicity of infection
NP: nucleoprotein
RING: really interesting new gene
RNP: ribonucleoprotein complex
TBK1: TANK-binding kinase 1
TRIM14: tripartite motif-containing 14.
