# Supplementary material for: Inhibition of Influenza A Virus Replication by TRIM14 via Its Multifaceted Protein–Protein Interaction With NP
Source: Front Microbiol. 2019 Feb 26;10:344. doi: 10.3389/fmicb.2019.00344 (PMC6401474; doi:10.3389/fmicb.2019.00344)
Supplement: Supplementary file 1 [file Data_Sheet_1.PDF]

Supplementary Material

Inhibition of influenza A virus replication by TRIM14 via its multifaceted protein-protein interaction with NP

Xiangwei Wu[1], Jingfeng Wang[1], Shanshan Wang[2], Fei Wu[1], Zhigao Chen[1] , Chunfeng Li[1], Genhong Cheng[1], F. Xiao-Feng Qin[1*]

[*]To whom correspondence should be addressed. Email: (F. Q.) fqin1@foxmail.com
Phone: (+86)-051262873785

Supplemental materials and methods

Primers of quantitative real-time PCR:

TRIM14-Forward:TGAAGGGGAAATTCACTGAACTC, Reverse: AGCCTCTGGACAGGATCGG.

HA-Forward: GAAGGAGGGCTCATACCCAAAGC, Reverse: CTGTTATAATTTGAGTCACTACA.

GAPDH-Forward: GAACGGGAAGCTCACTGG, Reverse: GCCTGCTTCACCACCTTCT.

IFNB-Forward: GATGAACTTTGACATCCCTGAG, Reverse: TCAACAATAGTCTCATTCCAGC;

ISG15-Forward: GAGAGGCAGCGAACTCATCT, Reverse: AGGGACACCTGGAATTCGTT;

Viperin-Forward: TGGGTGCTTACACCTGCTG, Reverse: GAAGTGATAGTTGACGCTGGTT;

IFIT1-Forward: TCAGGTCAAGGATAGTCTGGAG, Reverse: AGGTTGTGTATTCCCACACTGTA.

The sgRNA of target genes are as follows:

TRIM14:

sgRNA1-Forward: CACCGATCGTGTCAGGACAGCGT

sgRNA1-Reverse: AAACACGCTGGATCCTGACACGATC

sgRNA2-Forward: CACCGCATCGTGTCAGGATCCAGCG

sgRNA2-Reverse: AAACCGCTGGATCCTGACACGATGC

IFNAR1:

sgRNA1-Forward: CACCGGACCCTAGTGCTCGTCGCCG

sgRNA1-Reverse: AAACCGGCGACGAGCACTAGGGTCC

sgRNA2-Forward: CACCGGCACTAGGGTCGTCGCGCCC

sgRNA2-Reverse: AAACGGGCGCGACGACCCTAGTGCC

TBK1:

sgRNA1-Forward: CACCGGGAAATATCATGCGTGTTAT

sgRNA1-Reverse: AAACATAACACGCATGATATTTCC.

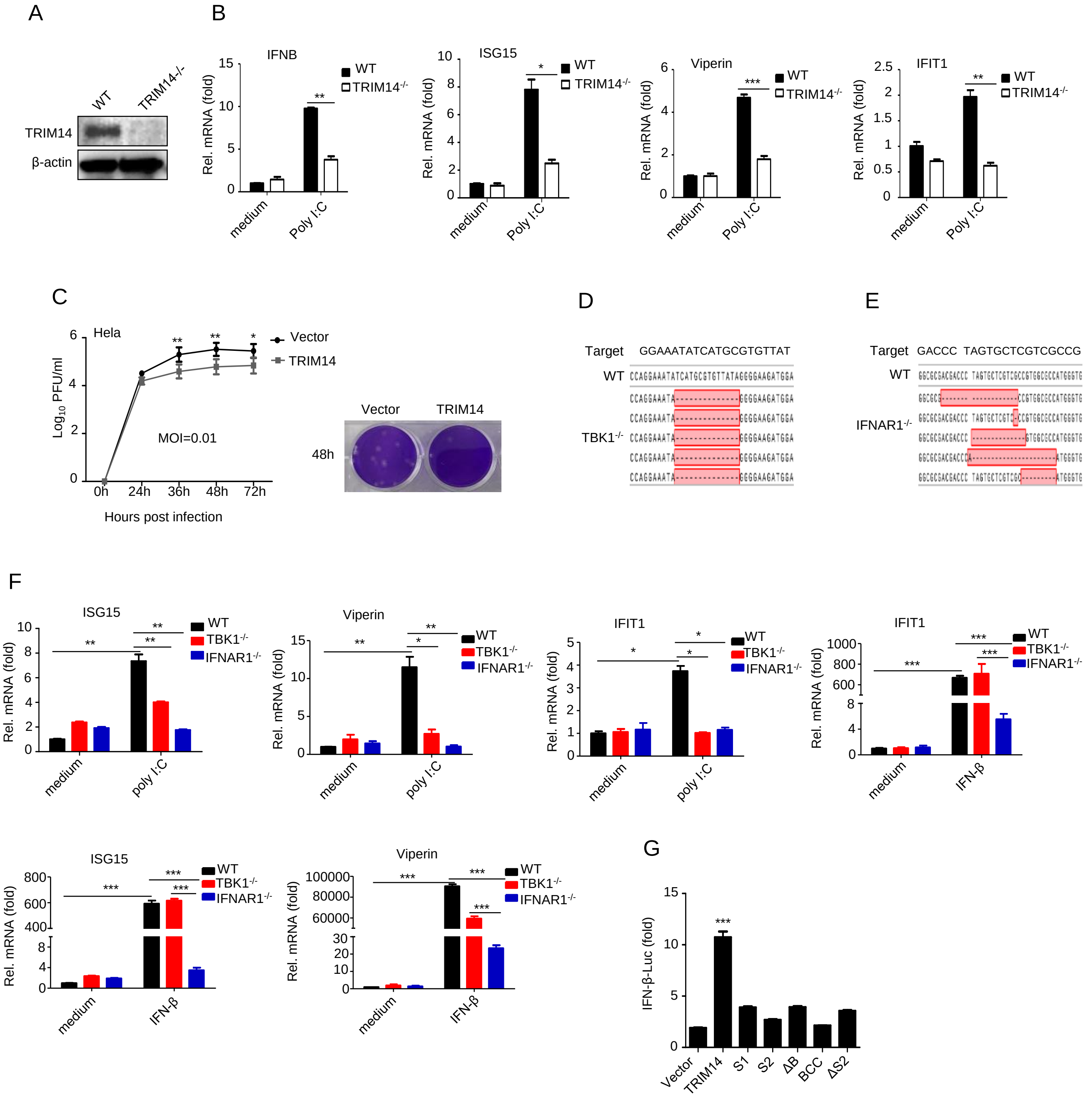

Figure S1, related to figure1. (A) Immunoblot analysis of TRIM14 in WT or TRIM14$^{-/-}$ HEK293T cells. (B) Real-time PCR analysis of HEK293T and TRIM14$^{-/-}$ HEK293T cells treated by 5μg/ml poly I:C. (C) Hela cells were transfected with HA-TRIM14 or a vector then cells were infected with WSN at an MOI of 0.01 at 24h post transfection. Viral titers were measured by plaque assay for the indicated time points. (D and E) Sequence alignment analysis of TBK1 knockout (D) and IFNAR1 knockout (E) HEK293T cells. (F) Real-time PCR analysis of ISGs mRNA expression in HEK293T, TBK1$^{-/-}$ HEK293T and IFNAR1$^{-/-}$ HEK293T cells. Cells were treated by 5μg/ml poly I:C or 100IU IFN-β. (G) Luciferase activity in HEK293T cells transfected with IFN-β luciferase reporter together with expression vectors of TRIM14 or TRIM14 mutants then cells were stimulated with SeV for 12h. Data are presented as mean ± SEM. * $P < 0.05$, ** $P < 0.01$, *** $P < 0.001$.

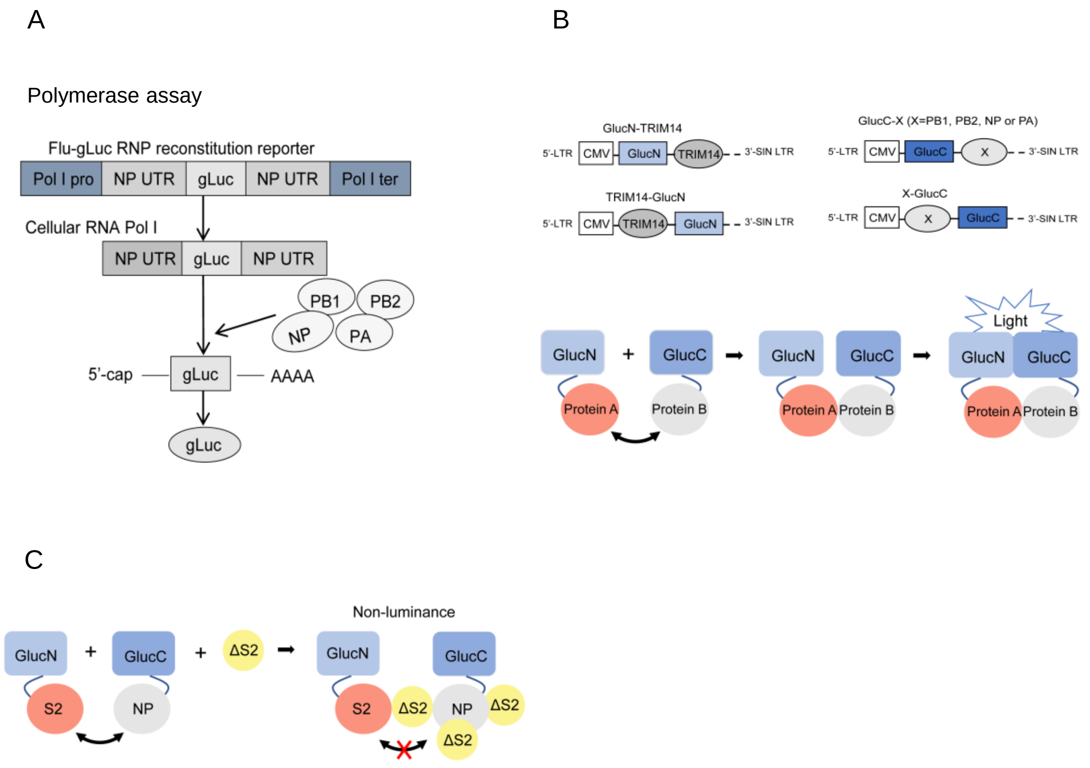

Figure S2 (A) Schematic diagram of IAV RNP reconstitution system. (B) Schematic depiction of GlucN-TRIM14/TRIM14-GlucN and GlucC-X/X-GlucC (X=PB1, PB2, PA, NP) used for BiLC system (top). The schematic illustration of protein interaction screened by BiLC assay (bottom). (C) The schematic illustration of effect of ΔS2 on S2-NP interaction screened by BiLC assay.

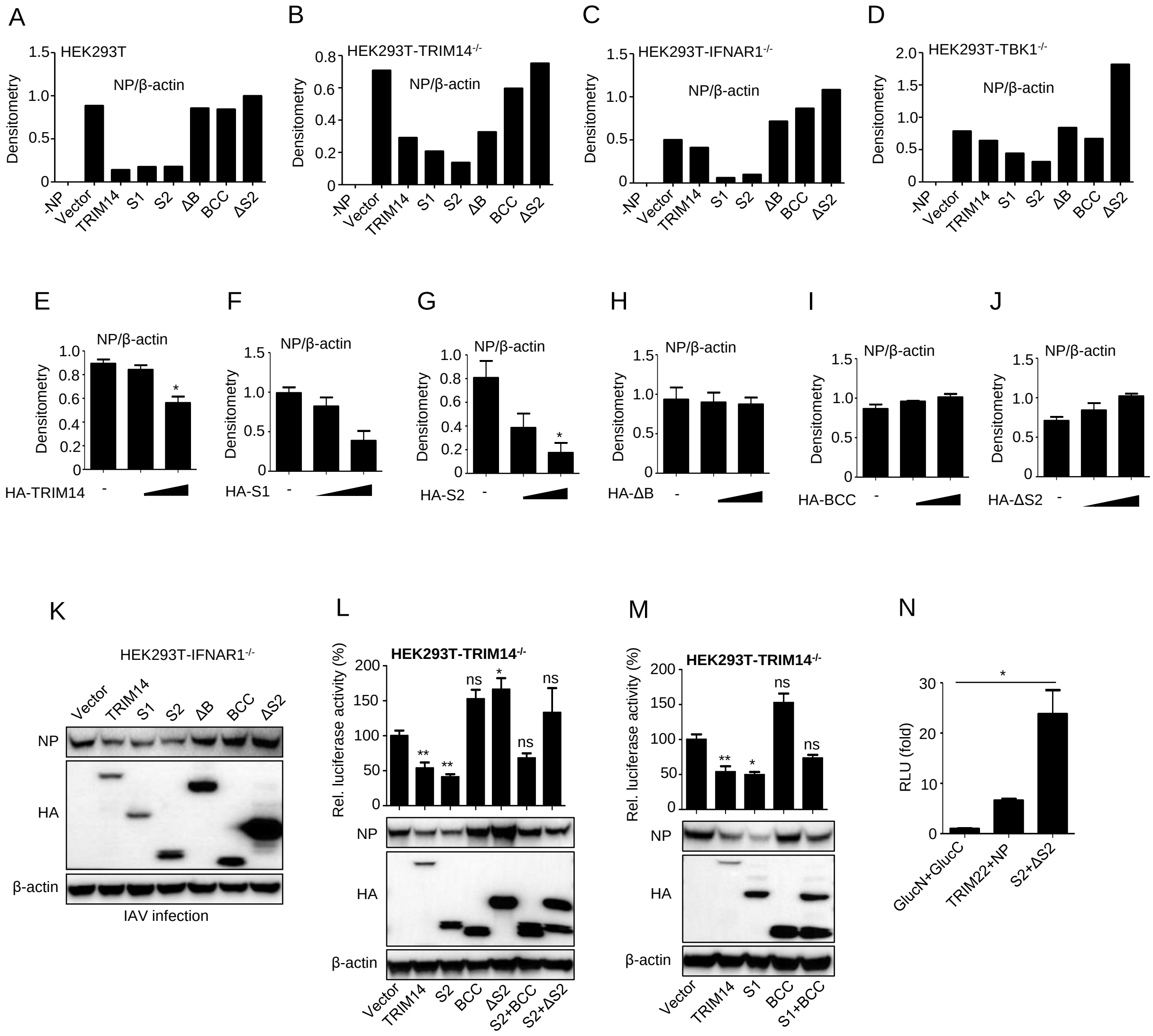

Figure S3, related to figure 4, 5. (A-D) The relative quantification of NP in figure 4A-D by using Image J software. (E-J) The relative quantification of NP in figure 4E-J by using Image J software. The results were presented as mean ± SEM of three independent experiments. (K) IFNAR1$^{-/-}$ HEK293T cells were infected with IAV-Luc after being transfected with TRIM14 or TRIM14 mutants, then cells were lysed for immunoassay at 24 hours post infection. (L) IAV RNP formation and immunoblot analysis of TRIM14$^{-/-}$ HEK293T cells cotransfected with RNP reconstitution reporter system and expression plasmids of TRIM14, S2, BCC, ΔS2, S2+BCC, S2+ΔS2. (M) IAV RNP formation and immunoblot analysis of TRIM14$^{-/-}$ HEK293T cotransfected with RNP reconstitution reporter system and expression plasmids of TRIM14, S1, BCC, S1+BCC. (N) The interaction between S2 and ΔS2 examined by BiLC assay. Data are presented as mean ± SEM. * $P < 0.05$, ** $P < 0.01$, ns: not significant.
